# Study on axial compressive properties of CFRP-confined ultra-high toughness cementitious composites

**DOI:** 10.1371/journal.pone.0331754

**Published:** 2025-09-25

**Authors:** Ban Li, Huang-kai Sun

**Affiliations:** 1 School of Architecture, Huanggang Polytechnic College, Huanggang, Hubei, China; 2 Institute of High Performance Engineering Structure, Wuhan University of Science and Technology, Wuhan, Hubei, China; Universiti Teknologi Malaysia, MALAYSIA

## Abstract

In order to study the mechanical properties of carbon fiber reinforced polymer (CFRP)-confined ultra-high toughness cementitious composites (UHTCC) under axial compression load, the preparation of UHTCC and the treatment of CFRP confinement were completed according to the specifications, considering the influence of single fiber types and CFRP confinement layers. After the axial compression load failure test, the failure phenomenon and stress-strain curves of the test were obtained. The influence mechanism of different single fiber types and different CFRP confinement layer on the axial compressive properties of CFRP-confined UHTCC was analyzed, and the stress-strain curves of the test was compared with the existing stress-strain models. The research results show that the ultimate compressive strength of UHTCC with steel fibers is significantly improved, reaching 84.6 MPa (compared to 27.6 MPa for unconfined specimens); The ultimate strain of UHTCC with PVA or PE increased significantly, with an increase of 107.6% and 243.2% respectively compared to unconfined specimens. The deviation between the calculated results of the Samaam model and the experimental results is less than 10%, indicating that the use of the Samaam model to propose a mathematical model for the stress-strain of CFRP-confined UHTCC is feasible.

## 1. Introduction

Ultra-high toughness cementitious composites (UHTCC) is composed of raw materials such as cement, fibers, and dispersing medium. It has been widely applied in bridges, highways, airport runways, oil and gas pipelines due to its high strength, high toughness, excellent wear resistance, corrosion resistance, as well as superior durability and chemical stability [1]. During service life, UHTCC structures are prone to varying degrees of damage caused by loading, adverse environmental conditions, and prolonged service periods, thereby compromising structural durability. The repair of damaged areas in UHTCC structures has remained a key research focus in civil engineering [2].

In practical engineering, the damaged areas of structures often contain cracks and deformations. Traditional confinement methods, such as stirrups or steel tubes, are difficult to apply for subsequent repair and reinforcement of these uneven areas. In contrast, Fiber Reinforced Polymer (FRP) composites are highly flexible and can be easily adhered to damaged areas of buildings. Additionally, FRP have a high strength-to-weight ratio and excellent corrosion resistance, making them the ideal material for confinement [3]. Commonly used FRP include Carbon Fiber Reinforced Polymer (CFRP), Glass Fiber Reinforced Polymer (GFRP), Aramid Fiber Reinforced Polymer (AFRP), and Basalt Fiber Reinforced Polymer (BFRP). Research has found that the use of carbon fiber reinforced polymer (CFRP) for repairing damaged areas has a better effect [4]. Mohammedameen et al. investigated the mechanical properties and durability of CFRP and BFRP-confined UHTCC in marine environments [5]. Their results revealed that CFRP-confined specimens exhibited 78.3%–83.5% higher compressive strength than BFRP-confined counterparts at equivalent curing ages, demonstrating CFRP’s enhanced protective capability against seawater erosion. Liang et al. systematically analyzed the influence of CFRP wrapping layers and concrete strength on the load-bearing capacity of CFRP-confined concrete prisms [6]. A linear increase in ultimate bearing capacity was observed with additional CFRP confinement layers. Xie et al. conducted axial compression tests on nine CFRP-confined circular columns considering size effects [7]. The ultimate compressive strength increased by approximately 38% with single-layer confinement and 75% with triple-layer confinement, highlighting the critical role of optimal wrapping layer configuration. These findings collectively confirm CFRP’s dual benefits in enhancing UHTCC’s marine erosion resistance and addressing insufficient load-bearing capacity under axial compression. This necessitates comprehensive experimental investigations to: 1) Determine the optimal CFRP wrapping layers and fiber types for maximizing UHTCC’s axial compression performance; 2) Establish a mathematically rigorous stress-strain model for CFRP-confined UHTCC systems.

Three distinct specimen configurations were fabricated with controlled variables including CFRP confinement layers (unconfined (0-layer), single-layer, and triple-layer) and fiber types (PVA, PE, and steel fibers). Through systematic analysis of failure mechanisms, stress-strain relationships, ultimate stresses, and corresponding strains, this study comprehensively evaluates the axial compression behavior of CFRP-confined UHTCC. A practically applicable stress-strain constitutive model was subsequently developed, demonstrating strong correlation with experimental observations.

### 1.1. Experimental program

UHTCC, a composite material comprising fibers dispersed in cementitious matrices (cement paste, mortar, or concrete), was formulated with 2 vol% hybrid fibers including polyvinyl alcohol (PVA), polyethylene (PE), and steel fibers. The mix proportion followed cement ash:sand:water = 0.91:3.63:0.91:1.00 [8]. Specimens measuring 100 mm × 100 mm × 300 mm were fabricated in triplicate per group. CFRP sheets exhibited mechanical properties of 1.7% elongation at break, 0.111 mm thickness, 3710 MPa tensile strength, and 241 GPa elastic modulus, with full fiber material characteristics detailed in Table 1.

**Table 1:** Physical properties of fibers.

Fiber type	Diameter/μm	Length/mm	Density/g.cm^-3^	Compressive strength/MPa	Elasticity modulus/GPa
PVA	39	12	1.3	1620	42.8
PE	21	12	0.97	3000	85
Steel-fiber	1000	25	7.8	400	200

Axial compression tests were conducted using an electro-hydraulic servo universal testing machine at Wuhan University of Science and Technology’s Civil Engineering Laboratory. Monotonic loading was applied at 1.5 kN/s until specimen failure [9], while axial deformations were measured via linear variable differential transformers (LVDTs).

**Fig 1:**
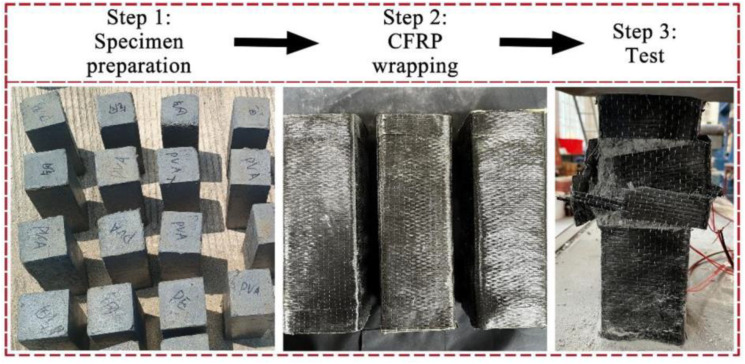
Experimental progress.

To ensure the fibers are evenly distributed, the cement, fly ash and sand were firstly mixed, and then the fibers were gradually added and mixed until a uniform state was achieved. Then water and superplasticizer were added and the mixture was vibrated at vibrating table until the material was well mixed. Finally, the mixture was placed into molds which was with oiled inner surfaces, and the specimens were demolded after 72 hours of curing [11]. All specimens were cured under conditions of (20 ± 2) °C and 95% humidity for 28 days [12], and all specimens were cast in the same batch. Then CFRP were wrapped around the specimens following the American Concrete Institute standards [13] in the following steps: first, the surface was cleaned to obtain a sound substrate; next, the epoxy resin was applied uniformly to the surface and finally, CFRP were then wrapped around the specimens. The experimental progress is shown in Fig 1.

## 2 Analysis of test results

### 2.1. Destructive phenomenon

(1) Failure mechanism analysis of unconfined specimen

**Fig 2:**
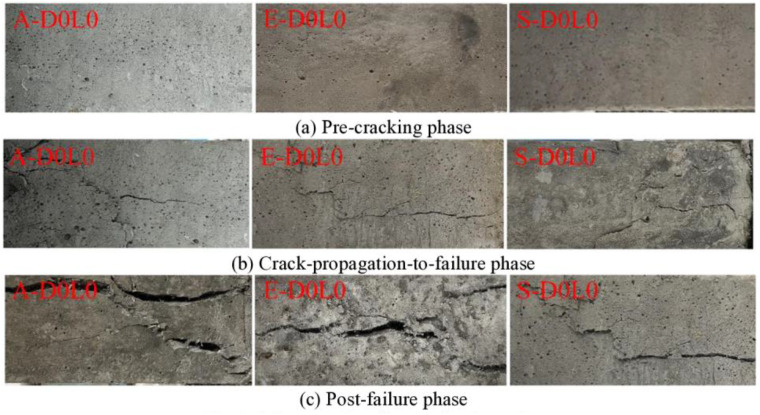
Failure modes of unconfined specimen. **(a)** Pre-cracking phase; **(b)** Crack-propagation-to-failure phase; **(c)** ‌Post-failure phase.

The failure progression of specimens A-D0L0 to S-D0L0 was categorized into three distinct phases: pre-cracking, crack-propagation, and post-failure, as schematically illustrated in Fig 2. Phase-contrast imaging (Fig 2a) revealed uniform apparent pore distribution and progressive color darkening across all specimens during pre-cracking. Crack-initiation patterns diverged significantly in the crack-propagation phase (Fig 2b): specimens A-D0L0 and S-D0L0 exhibited dominantly diagonal cracks originating from one end, while E-D0L0 developed predominantly vertical cracks. Post-failure quantification (Fig 2c) demonstrated substantial crack-width disparities-specimens A-D0L0 and E-D0L0 showed maximum vertical crack widths of 2.3 mm and 1.8 mm respectively, with A-D0L0 displaying 1.5 mm diagonal cracks. In contrast, S-D0L0 maintained narrower cracks (vertical: 0.9 mm, diagonal: 0.7 mm), substantiating the superior bridging effects of PVA and steel fibers in enhancing UHTCC’s fracture toughness. These observations align with the fiber-matrix interaction mechanisms reported by Zhou et al. [10].

(2) Failure mechanism analysis of CFRP-confined specimen

**Fig 3:**
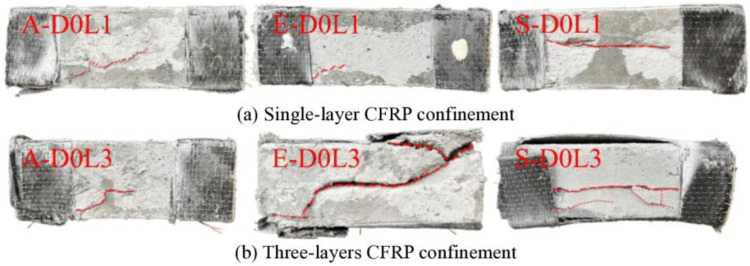
Failure modes of CFRP-confined specimen. **(a)** ‌Single-layer CFRP confinement; **(b)** Three-layers CFRP confinement.

The failure patterns of specimens A-D0L1 to S-D0L3 under axial compression are systematically compared with unconfined cases (Fig 3 vs Fig 2c). Enhanced CFRP confinement significantly suppressed crack development across all specimens except E-D0L3, with progressive reduction in crack propagation metrics (width, length, density) corresponding to increased CFRP layers. Specimens A-D0L1 and A-D0L3 demonstrated optimal CFRP confinement efficiency, followed by E-D0L1 and S-D0L1, while E-D0L3 exhibited minimal confinement effectiveness. This hierarchy confirms that UHTCC containing PE/steel fibers achieves peak axial compression performance under single-layer CFRP confinement, whereas PVA-reinforced UHTCC exhibits progressive enhancement with additional CFRP layers. The CFRP system provided substantial lateral confinement, increasing the ductility index by 18% ~ 35% compared to unconfined specimens through two mechanisms.

### 2.2 Analysis of ‌stress-strain curve

(1) The influence of ‌CFRP confinement layer

**Fig 4:**
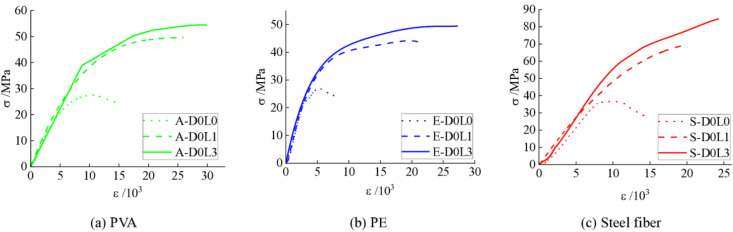
Stress-strain curves of different CFRP-confined layers. **(a)** PVA; **(b)** PE; **(c)** Steel fiber.

Fig 4 presents the stress-strain curves of specimens confined with varying CFRP layers. As illustrated, both the ultimate compressive strength and strain exhibit marked enhancements with increasing CFRP confinement layers. The UHTCC reinforced with PVA demonstrates intermediate improvements in ultimate strength and strain between PE-reinforced UHTCC and steel fiber-reinforced UHTCC (Fig 4a). Notably, the PE-reinforced UHTCC displays the smallest strength gain but the largest strain improvement, with specimen E-D0L3 achieving a 243% increase in ultimate strain compared to E-D0L0 (Fig 4b). For steel fiber-reinforced specimens, specimen S-D0L3 exhibits a 2.3-fold enhancement in ultimate compressive strength relative to S-D0L0 (Fig 4c). However, the limited deformation capacity of steel fibers leads to interfacial void formation between fibers and the cementitious matrix during loading. These voids hinder further stress redistribution, resulting in constrained ultimate strain improvements despite CFRP confinement. The steel fiber-reinforced system demonstrates the most rapid strength gain, characterized by the steepest post-peak slope in its stress-strain curve.

(2) ‌Influence of fiber type

**Fig 5:**
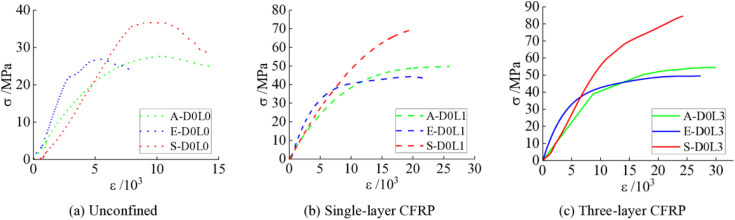
Stress-strain curves of different fiber types. **(a)** Unconfined; **(b)** ‌Single-layer CFRP; **(c)** Three-layer CFRP.

Fig 5 illustrates the stress-strain curves of specimens reinforced with different fiber types. The measured average compressive strengths and axial strains are summarized in Table 2. As shown in Fig 5a and Table 2, specimens A-D0L0 and S-D0L0 exhibit 1.85% and 32.84% increases in average compressive strength, along with 92.45% and 71.09% enhancements in average axial strain, respectively, compared to reference specimen E-D0L0. These results confirm the significant performance improvements imparted by steel and PVA fibers in UHTCC systems. From Figs 5b and 5c, the PE-reinforced UHTCC demonstrates higher initial load-bearing capacity, followed by gradual strength evolution with increasing strain. The PVA-reinforced UHTCC exhibits a delayed yield plateau compared to PE-reinforced UHTCC but precedes the steel fiber-reinforced system, ultimately achieving superior ultimate deformation capacity-indicating optimal ductility among the three systems. In contrast, the steel fiber-reinforced UHTCC lacks a distinct yield plateau, suggesting insufficient activation of the steel fibers by the cementitious matrix. This behavior, however, does not conclusively indicate inferior performance compared to PVA-reinforced UHTCC, as steel fibers primarily enhance strength rather than ductility.

**Table 2:** The dimensions and test results of the specimens.

Specimen		Dimensions	Fiber type	CFRP-layers	Averager compressive strength/MPa	Averager axial strain/μɛ
A-D0L0	1	100 mm × 100 mm × 300 mm	PVA	–	27.6	3135
	2	100 mm × 100 mm × 300 mm	PVA	–		
	3	100 mm × 100 mm × 300 mm	PVA	–		
E-D0L0	1	100 mm × 100 mm × 300 mm	PE	–	27.1	1629
	2	100 mm × 100 mm × 300 mm	PE	–		
	3	100 mm × 100 mm × 300 mm	PE	–		
S-D0L0	1	100 mm × 100 mm × 300 mm	Steel-fiber	–	36.0	2787
	2	100 mm × 100 mm × 300 mm	Steel-fiber	–		
	3	100 mm × 100 mm × 300 mm	Steel-fiber	–		
A-D0L1	1	100 mm × 100 mm × 300 mm	PVA	1	49.7	7782
	2	100 mm × 100 mm × 300 mm	PVA	1		
	3	100 mm × 100 mm × 300 mm	PVA	1		
E-D0L1	1	100 mm × 100 mm × 300 mm	PE	1	43.5	6475
	2	100 mm × 100 mm × 300 mm	PE	1		
	3	100 mm × 100 mm × 300 mm	PE	1		
S-D0L1	1	100 mm × 100 mm × 300 mm	Steel-fiber	1	69.6	6218
	2	100 mm × 100 mm × 300 mm	Steel-fiber	1		
	3	100 mm × 100 mm × 300 mm	Steel-fiber	1		
A-D0L3	1	100 mm × 100 mm × 300 mm	PVA	3	54.3	8991
	2	100 mm × 100 mm × 300 mm	PVA	3		
	3	100 mm × 100 mm × 300 mm	PVA	3		
E-D0L3	1	100 mm × 100 mm × 300 mm	PE	3	49.5	8192
	2	100 mm × 100 mm × 300 mm	PE	3		
	3	100 mm × 100 mm × 300 mm	PE	3		
S-D0L3	1	100 mm × 100 mm × 300 mm	Steel-fiber	3	84.6	7285
	2	100 mm × 100 mm × 300 mm	Steel-fiber	3		
	3	100 mm × 100 mm × 300 mm	Steel-fiber	3		

## 3 Proposal and Validation of a modified stress-strain model for CFRP-confined UHTCC

**Fig 6:**
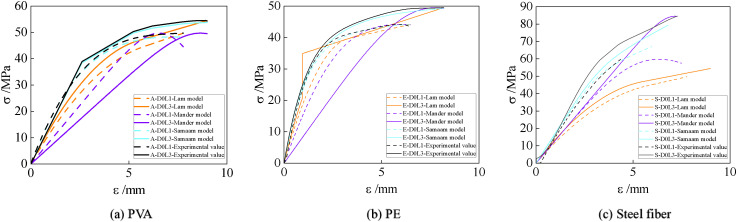
Comparison between calculation results and experimental results. **(a)** PVA; **(b)** PE; **(c)** Steel fiber.

To establish a mathematical model for the stress-strain behavior of CFRP-confined UHTCC, the predictions of the Lam model [11], Mander model [12], and Samaam model [13] were compared with experimental results, as illustrated in Fig 6. As shown in Figs 6a and 6b, the Lam model aligns closely with the test data for PVA- and PE-confined UHTCC but exhibits significant deviations for steel fiber-reinforced UHTCC. Specifically, the Lam model underestimates the CFRP-induced enhancement in ultimate compressive strength and overestimates its deformation capacity. Fig 6c reveals that the Mander model yields conservative predictions, particularly underestimating the elastic modulus during the elastic phase. While the Mander model shows acceptable accuracy for single-layer CFRP confinement (error <8%), its predictions deviate markedly for triple-layer CFRP confinement (error >25%). Overall, the Samaam model demonstrates superior agreement with experimental results across all fiber types and CFRP configurations, justifying its adoption for developing the stress-strain model for CFRP-confined UHTCC.

The Samaam model divides the compression process of CFRP-confined concrete into two phases-elastic and strengthening-while introducing an additional curve-shape control parameter to ensure smooth transition between phases [13], which aligns with the observed mechanical response. During the ‌elastic phase‌, the axial load is relatively low, and the specimen resists external forces primarily through its intrinsic strength, resulting in minimal deformation. In this phase, the elastic moduli of CFRP-confined and unconfined specimens differ marginally. In the ‌strengthening phase‌, as stress increases beyond the specimen’s inherent capacity, lateral expansion initiates. The CFRP confinement effectively suppresses this expansion, enabling synergistic load sharing between the CFRP and concrete matrix. Furthermore, the high compressive strength and elastic modulus of CFRP significantly enhance the ultimate strain and stress of confined specimens. The strengthening phase exhibits a gradual slope variation and stable deformation, consistent with the model’s predictions.

**Fig 7:**
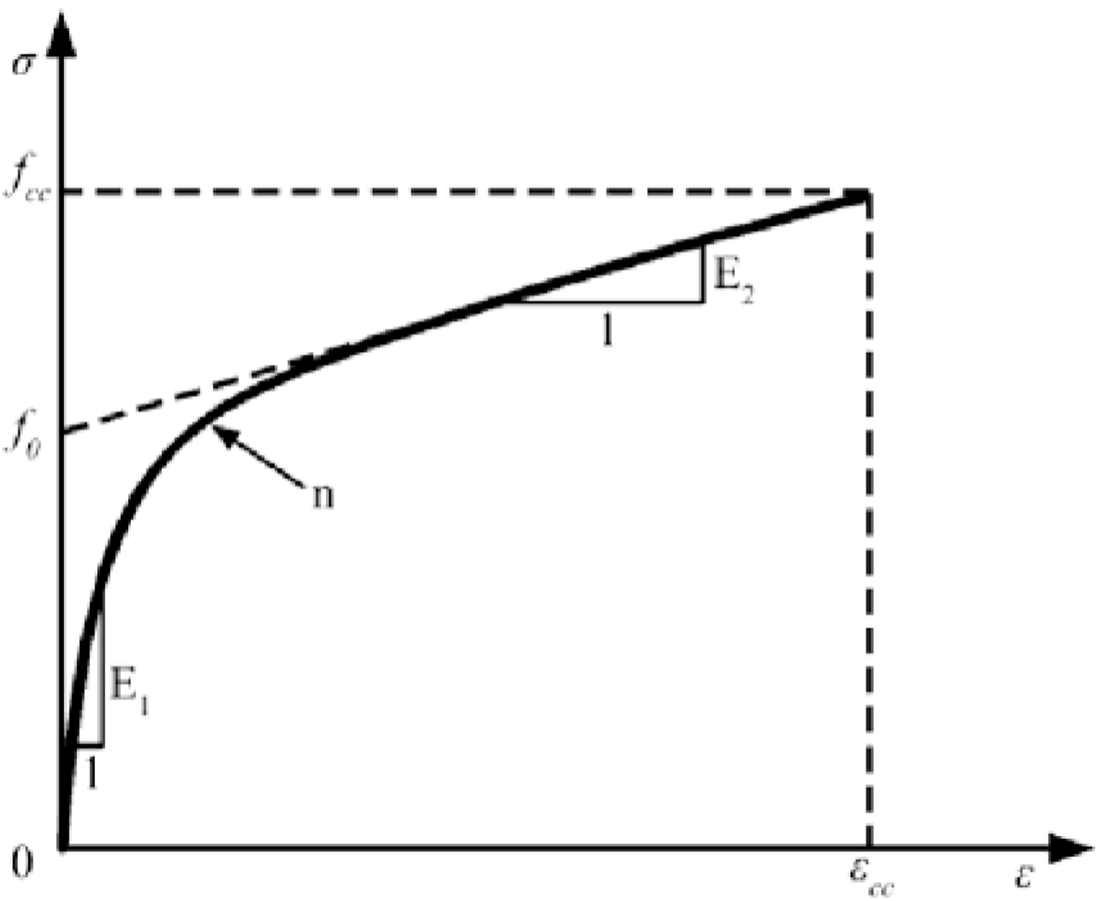
Diagram of Samaam Model.

The constitutive equation of the Samaam model is formulated as Eq. (1), which divides the stress-strain response of CFRP-confined UHTCC into distinct elastic and strengthening phases while incorporating a curve-shape control parameter to ensure continuity between phases [13].


$$\sigma =\frac{(E_{1}-E_{2})\varepsilon_{c}}{{\left[1+{\left(\frac{(E_{1}-E_{2})\varepsilon_{c}}{f_{0}}\right)}^{n}\right]}^{1/n}}+E_{2}\varepsilon$$ (1)

where *n* is a dimensionless curve shape control parameter. *f*_*0*_ is the intercept of the reverse extension line of the second straight segment on the stress axis, which is related to the number and type of CFRP constraint layers, as well as the constraint stress. *E*_*1*_ and *E*_*2*_ are the slopes of the first and second straight line segments, respectively, calculated from Fig 7.

**Fig 8:**
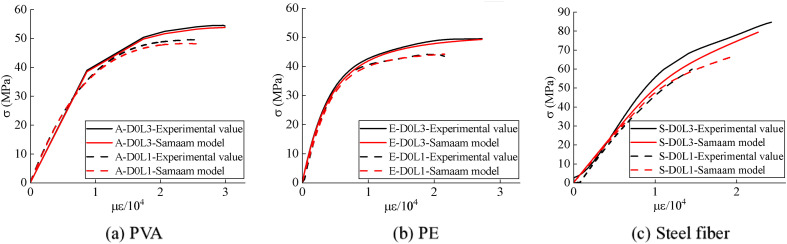
Comparison between Samaam model and experimental results. **(a)** PVA; **(b)** PE; **(c)** Steel fiber.

This study conducted experimental investigations on the axial compressive behavior of CFRP-confined UHTCC, evaluating the effects of CFRP confinement layers and fiber types (PVA, PE, steel). The optimal configuration for maximizing compressive performance was identified as ‌steel fiber-reinforced UHTCC with triple-layer CFRP confinement‌. A constitutive stress-strain model for CFRP-confined UHTCC was subsequently developed based on the Samaam framework (Fig 8).

The computational results of the Samaam model and corresponding experimental data are summarized in Table 3. The proposed ‌constitutive stress-strain model‌ for CFRP-confined UHTCC, derived from the Samaam framework, exhibits deviations of ‌less than 10%‌ compared to experimental measurements.

**Table 3:** Comparison between the results of Samaam model and experimental results.

Fiber type	Mean	SD	MAE
PVA	0.98	0.01	0.02
PE	0.99	0.02	0.01
Steel-fiber	1.03	0.05	0.03

## 4 Conclusion

This article collects the failure phenomenon and experimental data of CFRP-confined UHTCC under axial compression, revealing the influence of different layers of CFRP confinements and different fiber types. With the help of three current stress-strain mathematical models, the Samaam model is the proposed model for CFRP constrained UHTCC stress-strain is proposed. The research results are as follows:

(1) The number of CFRP confinement layers has a significant impact on the compressive performance of UHTCC. As the number of CFRP confinement layers increases, the ultimate compressive strength and ultimate deformation of the specimen both increase, exhibiting good ductility performance. When 3 layers of CFRP are added, the confinement effect is optimal, and the compressive strength of UHTCC mixed with PVA, PE, and steel fibers is increased by 68.8%, 57.4%, and 96.2%, respectively.
(2) The influence of fiber types on the compressive performance of UHTCC cannot be ignored. Specifically, the compressive strength of UHTCC doped with steel fibers is significantly improved. The ultimate strain of UHTCC doped with PVA and PE was significantly increased, with an increase of 107.6% and 243.2% respectively compared to unconstrained UHTCC.
(3) The Samaam model is used to propose a mathematical model for CFRP-confined UHTCC stress-strain, which has good calculation accuracy and meets the requirements of this study.

## Supporting information

S1 File Study on axial compressive properties of CFRP-confined ultra-high toughness cementitious composites. (ZIP)
